# Comparison of Fractional Flow Reserve and Resting Full-Cycle Ratio in the Functional Assessment of Coronary Artery Stenosis in Patients with Non-ST-Segment Elevation Acute Coronary Syndrome

**DOI:** 10.31083/j.rcm2507260

**Published:** 2024-07-11

**Authors:** Yumeng Lei, Mao Jiang, Xu Liu, Shuaiyong Zhang, Mengyao Li, Yunfei Wang, Ming Chen, Nan Guo, Yongxing Liu, Xufen Cao, Liqiu Yan

**Affiliations:** ^1^Department of Cardiology, Dongguan Songshan Lake Central Hospital, Guangdong Medical University, 523109 Dongguan, Guangdong, China; ^2^Department of Cardiology, Cangzhou Central Hospital, Hebei Medical University, 061007 Cangzhou, Hebei, China

**Keywords:** fractional flow reserve, resting full-cycle ratio, acute coronary syndrome, functional assessment

## Abstract

**Background:**

This study investigated factors influencing discrepancies between fractional flow reserve (FFR) and resting full-cycle ratio (RFR) in the functional assessment of coronary artery stenosis in patients with non-ST-segment elevation acute coronary syndrome (NSTE-ACS).

**Methods:**

We included 320 diseased vessels from 253 consecutive patients with NSTE-ACS. Vessels were categorized into four groups based on FFR ≤0.80 and RFR ≤0.89 thresholds: group 1 concordant negative (RFR–/FFR–), group 2 positive RFR and negative FFR (RFR+/FFR–), group 3 negative RFR and positive FFR (RFR–/FFR+), and group 4 concordant positive (RFR+/FFR+). Univariate and multivariate logistic regression analyses were conducted to identify predictors of diagnostic discrepancy between FFR and RFR.

**Results:**

Of the 320 diseased vessels, 182 (56.9%) were in group 1 (RFR–/FFR–), 33 (10.3%) in group 2 (RFR+/FFR–), 31 (9.7%) in group 3 (RFR–/FFR+), and 74 (23.1%) in group 4 (RFR+/FFR+). The concordance between FFR and RFR was 80.0%. Notably, left anterior descending artery (LAD) lesions exhibited significantly lower consistency compared to non-LAD lesions (*p* = 0.001), with distinct differences in FFR and RFR values between these groups (*p* < 0.001). The presence of a LAD lesion emerged as an independent predictor of diagnostic inconsistency between positive RFR and negative FFR measurements (*p* = 0.001).

**Conclusions:**

LAD involvement independently predicts diagnostic discrepancies between FFR and RFR in evaluating functional coronary artery stenosis in NSTE-ACS patients.

## 1. Introduction

The fractional flow reserve (FFR) is an invasive physiological index used to evaluate the impact of coronary artery stenosis on myocardial ischemia, thus guiding the revascularization strategy. Multiple studies have shown that FFR–guided percutaneous coronary intervention (PCI) outperforms angiography-guided PCI [1, 2, 3]. Despite its effectiveness, FFR measurement necessitates the administration of vasodilators to induce maximum hyperemia. This requirement is linked with several disadvantages, including extended procedure times, potential side effects from the drugs, and patient discomfort. These drawbacks have contributed to FFR’s relatively low clinical adoption rate of 6–8% [4].

In response to these challenges, the development of non-hyperemic pressure ratios (NHPRs), such as instantaneous wave-free ratio (iFR) and diastolic pressure ratio, has significantly alleviated these limitations. Two large-scale randomized controlled trials demonstrated that the 1-year clinical outcomes of revascularization strategies guided by iFR and those guided by FFR are comparable [3, 5]. Additionally, the resting full-cycle ratio (RFR) is a novel NHPR approach used to identify the lowest value of the ratio of distal coronary artery pressure to aortic artery pressure over five consecutive complete heart cycles. Research indicates that RFR’s diagnostic efficacy is on par with that of iFR, offering a comprehensive assessment by incorporating both diastolic and systolic phases of the cardiac cycle [6].

While international studies have shown strong consistency between FFR and RFR [7, 8, 9, 10], research focusing on the discrepancy between FFR and RFR in Chinese patients is limited. This study aimed to identify factors influencing the inconsistency between FFR and RFR in the functional assessment of coronary artery stenosis in Chinese patients with non-ST-segment elevation acute coronary syndrome (NSTE-ACS).

## 2. Materials and Methods

### 2.1 Study Participants

This single-center, prospective registry study was conducted at the Cangzhou Central Hospital of Hebei Medical University. We enrolled 267 patients (representing a total of 337 diseased vessels) with NSTE-ACS, who underwent coronary angiography (CAG) and invasive coronary artery functional assessment between September 2021 and August 2023. The inclusion criteria were as follows: (1) age ≥18 years; (2) diagnosis of NSTE-ACS; (3) CAG showing stenosis severity ranging from 30 to 90%; and (4) voluntary agreement to undergo invasive coronary functional assessment. NSTE-ACS is defined as [11] acute chest discomfort with positive cardiac biomarkers but no continuous ST-segment elevation that may include transient ST-segment elevation, continuous or transient ST-segment depression, or T-wave inversion and low T-wave. Moreover, NSTE-ACS can be classified into non-ST-segment elevation myocardial infarction (NSTEMI) and unstable angina (UA) based on myocardial necrosis biomarkers. The exclusion criteria were as follows: (1) severe bronchial asthma or intolerance to vasodilators like adenosine; (2) atrioventricular block of second-degree or worse; (3) cardiogenic shock; and (4) absence of concurrent RFR and FFR measurement or data drift. Based on the exclusion criteria, 14 patients (17 diseased vessels), including 13 not assessed for RFR and 1 with FFR data drift, were excluded. Consequently, 253 patients (representing a total of 320 vessels) were included in the analysis. The study protocol was approved by the Ethics Committee of Cangzhou Central Hospital of Hebei Medical University, and informed consent was obtained from all participants.

### 2.2 Functional Assessment of Coronary Artery

All participants underwent CAG via the radial artery approach. The decision to proceed with invasive functional assessment was made by two skilled interventional cardiologists based on the outcomes of the CAG and the patient’s clinical status. The following situations required FFR/RFR measurements: (1) the presence of coronary artery stenosis through CAG but difficulty in determining whether PCI was needed; (2) the presence of branch lesions; and (3) the presence of symptoms of myocardial ischemia (such as angina) but with normal or unexplained CAG results. Before the assessment, intracoronary nitroglycerin (200 µg) was administered as a routine measure to avert coronary artery spasm. A pressure guidewire, PressureWire™ x0.014 (Abbott Vascular Inc., Santa Clara, CA, USA), was positioned in the distal segment of the lesion. The RFR value was initially recorded in a non-hyperemic state. Following this, adenosine triphosphate disodium (167 µg/kg/min) was infused intravenously through the median cubital vein to induce maximal hyperemia, and the FFR value was measured. Notably, failure to induce maximum congestion may lead to an overestimation of FFR and an underestimation of the true severity of the lesion. Thus, the dose of adenosine triphosphate disodium was 167 µg/kg/min in this study. An FFR ≤0.80 is considered the gold standard for diagnosing functional ischemia of the coronary artery. In this study, the final revascularization plans for patients were completely dependent on the results of the FFR measurements.

### 2.3 Statistical Analysis

Continuous variables were assessed for normality using the Kolmogorov–Smirnov test. Normally distributed variables are presented as means ± standard deviations, whereas those not distributed normally are presented as medians and interquartile ranges. Categorical variables are expressed as frequencies (percentages). Depending on the data distribution, continuous variables were analyzed using the student *t*-test or the Mann–Whitney U test. Categorical variables were compared using the chi-square test or Fisher exact test, as appropriate. For comparisons across more than two groups, the Kruskal–Wallis test was employed. Subsequent post-hoc analysis with the Mann–Whitney U test along with the Bonferroni correction was utilized for multiple comparisons of the baseline characteristics among the groups. Both univariate and multivariate logistic regression models were employed to identify predictors of discrepancies between FFR and RFR. Variables with *p*-values ≤ 0.10 in the univariate analysis were subsequently included in the multivariate logistic regression model. Two-sided *p*-values < 0.05 were considered statistically significant. All statistical analyses were conducted using Statistical Product and Service Solutions software, version 25.0 (IBM Corporation, Armonk, NY, USA).

## 3. Results

### 3.1 Baseline Characteristics

The study cohort included 253 patients, of which 41.5% were female, and the median age was 62.4 (56.5–69.0) years. NSTEMI was present in 5.9% of the cases. A significant portion of the study population had comorbid conditions: 58.9% of the patients had hypertension, 24.5% had diabetes, and 12.6% had a history of smoking (Table 1).

**Table 1.** **Baseline clinical characteristics**.

Characteristics		Patients (n = 253)
Age, years		62.4 (56.5–69.0)
Female sex		105 (41.5%)
BMI, ${\mathrm{kg/m}}^{2}$		25.4 (23.5–27.3)
Hypertension		149 (58.9%)
Dyslipidemia		36 (14.2%)
Diabetes		62 (24.5%)
Smoking		32 (12.6%)
Drinking		23 (9.1%)
Previous AMI		6 (2.4%)
Previous PCI		23 (9.1%)
Previous stroke		22 (8.7%)
Atrial fibrillation		5 (2.0%)
Peripheral vascular disease		2 (0.8%)
Creatinine, µmol/L		64.2 (54.0–71.0)
Hematocrit, g/L		135.9 (128.0–143.5)
Hematocrit		40.9 (38.3–43.1)
Clinical presentation		
	Unstable angina	238 (94.1%)
	NSTEMI	15 (5.9%)

Values are presented as medians (interquartile range) or n (%). BMI, body mass index; AMI, acute myocardial infarction; PCI, percutaneous coronary intervention; NSTEMI, non-ST-segment elevation myocardial infarction.

**Fig. 1.**
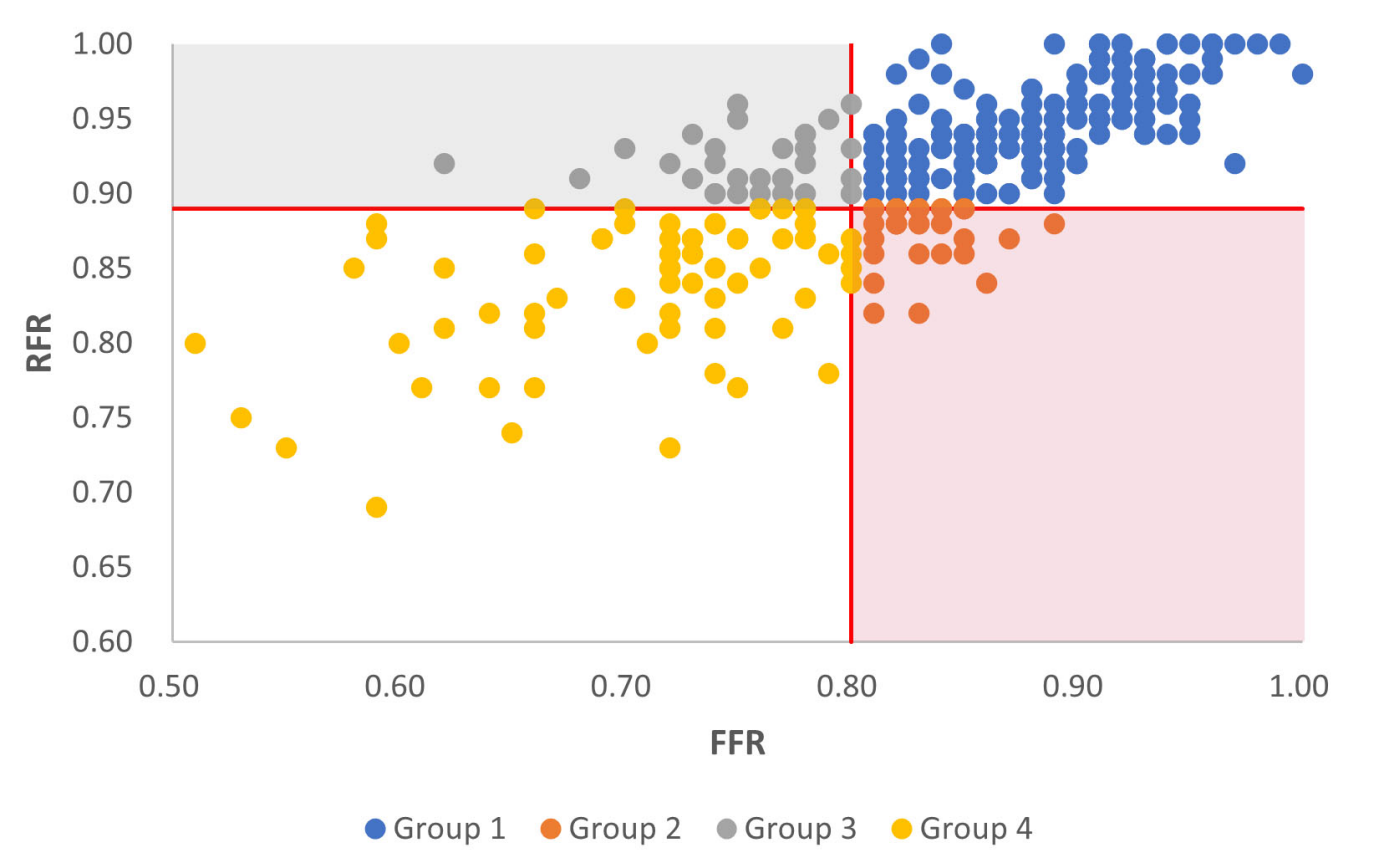
**Distribution of lesions according to the fractional flow reserve and resting full-cycle ratio values.** The lesions are classified according to the cutoff values of FFR ≤0.8 and RFR ≤0.89 as follows: group 1 (RFR–/FFR–): concordant negative, group 2 (RFR+/FFR–): positive RFR and negative FFR, group 3 (RFR–/FFR+): negative RFR and positive FFR, and group 4 (RFR+/FFR+): concordant positive. FFR, fractional flow reserve; RFR, resting full-cycle ratio.

All participants underwent CAG via the radial artery approach. Based on the cutoff values of FFR ≤0.8 and RFR ≤0.89, the diseased vessels were categorized into the following four groups: group 1 (RFR–/FFR–): concordant negative, group 2 (RFR+/FFR–): positive RFR and negative FFR, group 3 (RFR–/FFR+): negative RFR and positive FFR, and group 4 (RFR+/FFR+): concordant positive (Fig. 1).

**Fig. 2.**
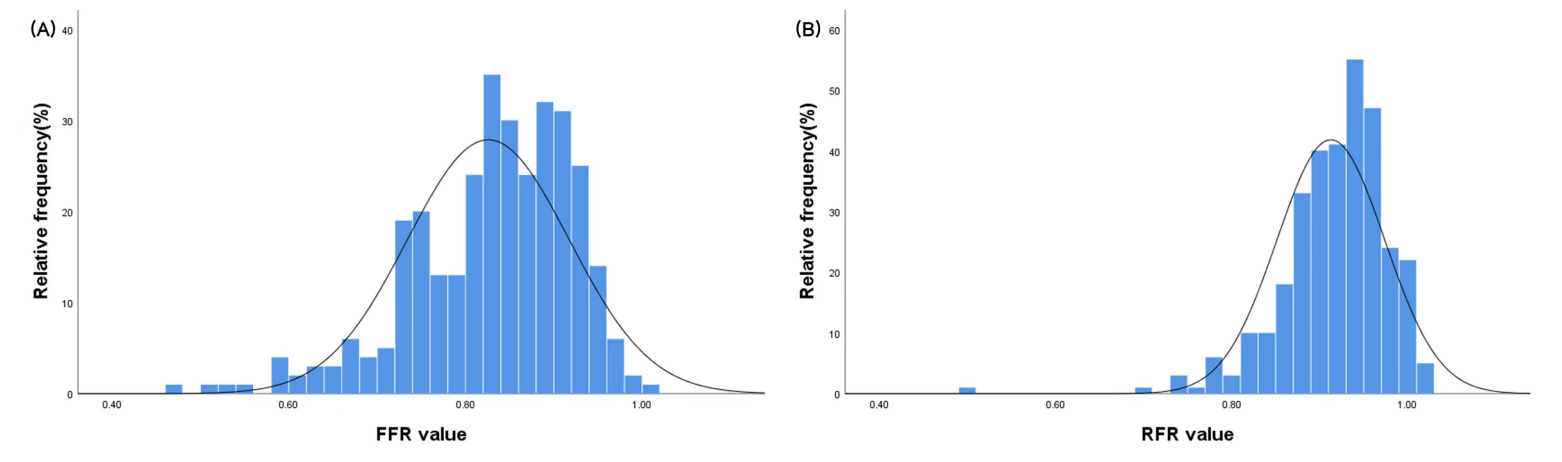
**Distributions of the fractional flow reserve and resting full-cycle ratio values.** (A) FFR. (B) RFR. FFR, fractional flow reserve; RFR, resting full-cycle ratio.

The distribution of the FFR and RFR values is depicted in Fig. 2. The median values of FFR and RFR were 0.83 (0.77–0.89) and 0.91 (0.88–0.95), respectively. Overall, the study examined 320 diseased vessels, finding that 89.7% exhibited a stenosis severity ≥70%. The left anterior descending artery (LAD) was the most common location for these lesions, accounting for 63.4% of the total, and 31.2% of the lesions received interventional therapy (Table 2).

**Table 2.** **Angiographic and physiological characteristics and treatment strategies**.

		Concordance (n = 256)	Discordance (n = 64)	All lesions (n = 320)
Location of diseased vessels				
	Left anterior descending artery	151 (59.0%)	52 (81.2%)	203 (63.4%)
	Left circumflex artery	38 (14.8%)	1 (1.6%)	39 (12.2%)
	Right coronary artery	67 (26.2%)	11 (17.2%)	78 (24.4%)
Angiographic stenosis				
	<50	3 (1.2%)	0	3 (0.9%)
	50–59%	7 (2.7%)	1 (1.6%)	8 (2.5%)
	60–69%	17 (6.6%)	5 (7.8%)	22 (6.9%)
	≥70%	229 (89.5%)	58 (90.6%)	287 (89.7%)
RFR		0.93 (0.88–0.96)	0.89 (0.88–0.92)	0.91 (0.88–0.95)
Resting Pd/Pa		0.95 (0.92–0.98)	0.93 (0.92–0.95)	0.94 (0.92–0.98)
FFR		0.86 (0.78–0.91)	0.81 (0.76–0.83)	0.83 (0.77–0.89)
Final treatment strategy				
	Interventional therapy	72 (28.1%)	28 (43.8%)	100 (31.2%)
	Medication	184 (71.9%)	36 (56.2%)	220 (68.8%)

Values are presented as medians (interquartile ranges) or n (%). RFR, resting full-cycle ratio; Pd, distal arterial pressure; Pa, arterial pressure within the entire cardiac cycle; FFR, fractional flow reserve.

### 3.2 Consistency Comparison between FFR and RFR

**Fig. 3.**
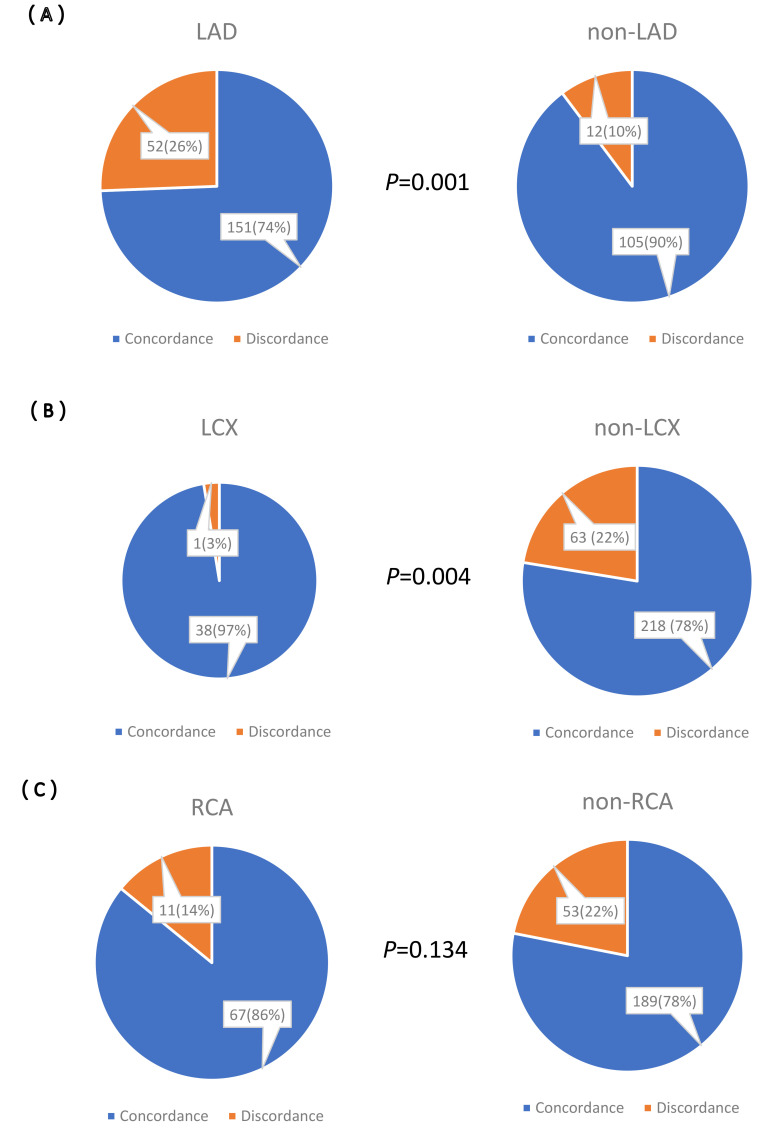
**Concordance distributions of resting full-cycle ratio and fractional flow reserve values of different vessels. **(A) LAD and non-LAD. (B) LCX and non-LCX. (C) RCA and non-RCA. LAD, left anterior descending artery; LCX, left circumflex artery; RCA, right coronary artery.

Among the 320 diseased vessels, a significant level of agreement between FFR and RFR values was observed in 80.0% of cases (n = 256 vessels). Specifically, of these concordant vessels, 203 lesions were located in the LAD and 117 in the non-LAD areas. The concordance rates for the LAD and non-LAD lesions were 74.0% (151/203) and 90.0% (105/117), respectively, with the LAD lesions showing significantly lower consistency than the non-LAD lesions (*p* = 0.001). The concordance rates for the LCX and non-LCX lesions were 97.0% (38/39) and 78.0% (218/281), respectively, with LCX lesions showing significantly higher consistency than the non-LCX lesions (*p* = 0.004). However, no significant difference in the rate of consistency was observed between the RCA and non-RCA lesions (*p* = 0.134) (Fig. 3).

A significant difference in the FFR and RFR values was observed between the LAD and non-LAD lesions (*p* < 0.001) (Table 3). Of the 320 diseased vessels, 182 (56.9%) belonged to group 1 (RFR–/FFR–), 33 (10.3%) to group 2 (RFR+/FFR–), 31 (9.7%) to group 3 (RFR–/FFR+), and 74 (23.1%) to group 4 (RFR+/FFR+). No significant differences were observed in age, sex, complications, and biochemical indices (hemoglobin and creatinine) among the four groups (*p* > 0.05). However, significant differences were noted in the LAD involvement and the degree of angiographic vascular stenosis among the four groups (*p* ≤ 0.001) (Table 4).

**Table 3.** **Fractional flow reserve and resting full-cycle ratio values of the left anterior descending artery, left circumflex artery, and right coronary artery**.

	LAD	LCX	RCA	*p-*value
FFR	0.81 (0.75–0.86)	0.89 (0.84–0.93)^a^	0.85 (0.78–0.93)^a^	<0.001
RFR	0.89 (0.87–0.93)	0.96 (0.94–0.98)^b^	0.94 (0.93–0.98)^b^	<0.001

Values are presented as medians (interquartile ranges) or n (%). FFR, fractional flow reserve; RFR, resting full-cycle ratio; LAD, left anterior descending artery; LCX, left circumflex artery; RCA, right coronary artery. ^a^*p* < 0.001 for FFR of LAD compared with FFR of LCX and RCA. ^b^*p* < 0.001 for RFR of LAD compared with FFR of LCX and RCA.

**Table 4.** **Clinical, angiographic, and physiologic characteristics of the four groups classified by the cutoff values of the fractional flow reserve and resting full-cycle ratio**.

	Group 1 (n = 182) (RFR >0.89, FFR >0.80)	Group 2 (n = 33) (RFR ≤0.89, FFR >0.80)	Group 3 (n = 31) (RFR >0.89, FFR ≤0.80)	Group 4 (n = 74) (RFR ≤0.89, FFR ≤0.80)	*p*-value
Age, years	63.4 (57.8–69.0)	63.1 (57.5–68.0)	58.8 (52.0–66.0)	62.0 (56.0–69.0)	0.072
Female sex	72 (39.6%)	19 (57.6%)	12 (38.7%)	31 (41.9%)	0.276
BMI, ${\mathrm{kg/m}}^{2}$	25.1 (23.5–26.4)	25.7 (24.6–27.8)	26.0 (24.2–28.2)	25.7 (23.8–27.8)	0.059
Hypertension	106 (58.2%)	21 (63.6%)	17 (54.8%)	45 (60.8%)	0.883
Dyslipidemia	27 (14.8%)	3 (9.1%)	9 (29.0%)	13 (17.6%)	0.149
Diabetes	44 (24.2%)	9 (27.3%)	7 (22.6%)	16 (21.6%)	0.929
Smoking	31 (17.0%)	1 (3.0%)	5 (16.1%)	8 (10.8%)	0.127
Drinking	22 (12.1%)	1 (3.0%)	4 (12.9%)	5 (6.8%)	0.290
Previous AMI	3 (1.6%)	2 (6.1%)	0	3 (4.1%)	0.213
Previous PCI	17 (9.3%)	4 (12.1%)	1 (3.2%)	7 (9.5%)	0.669
Previous stroke	18 (9.9%)	2 (6.1%)	2 (6.5%)	5 (6.8%)	0.865
Atrial fibrillation	6 (3.3%)	0	0	0	0.398
Peripheral vascular disease	3 (1.6%)	0	1 (3.2%)	0	0.365
Creatinine, µmol/L	65.4 (56.0–74.0)	59.9 (50.0–69.0)	66.5 (58.0–71.0)	62.2 (52.0–70.3)	0.149
Hematocrit, g/L	135.3 (127.0–141.3)	135.0 (128.0–141.5)	139.4 (130.0–149.0)	135.3 (128.8–145.0)	0.414
Hematocrit	40.6 (38.1–42.3)	40.5 (38.0–42.3)	41.5 (39.6–43.7)	40.8 (38.1–43.3)	0.422
LAD lesion	92 (50.5%)	32 (97.0%)	20 (64.5%)	59 (79.7%)	<0.001
Angiographic stenosis	74.0 (70.0–80.0)	74.9 (70.0–80.0)	80.3 (80.0–85.0)	81.8 (80.0–85.0)	<0.001

Values are presented as medians (interquartile ranges) or n (%). BMI, body mass index; AMI, acute myocardial infarction; PCI, percutaneous coronary intervention; LAD, left anterior descending artery; FFR, fractional flow reserve; RFR, resting full-cycle ratio.

### 3.3 Factors Influencing the Diagnostic Inconsistency between FFR and RFR

The univariate logistic regression analysis identified several factors with discordance between RFR and FFR measurements. Specifically, sex, a history of smoking, creatinine levels, and LAD location (each with a *p*-value ≤ 0.10) were associated with a discordance where RFR was ≤0.89 and FFR was >0.80 (Table 5). Similarly, age and LAD location (*p*-values ≤ 0.10) were associated with a discordance where RFR was >0.89 and FFR was ≤0.80 (Table 6).

**Table 5.** **Independent predictors of discordance between resting full-cycle ratio ≤0.89 and fractional flow reserve >0.80**.

	Univariate analysis			Multivariate analysis		
	OR	95% CI	*p-*value	OR	95% CI	*p-*value
Age, years	0.996	0.955–1.040	0.859			
Female sex	2.073	0.978–4.396	0.057	1.115	0.410–3.034	0.832
BMI, ${\mathrm{kg/m}}^{2}$	1.067	0.942–1.209	0.309			
Hypertension	0.797	0.370–1.718	0.563			
Dyslipidemia	1.742	0.496–6.112	0.386			
Diabetes	0.850	0.368–1.965	0.704			
Smoking	6.570	0.865–49.897	0.069	6.914	0.872–54.802	0.067
Drinking	4.400	0.572–33.826	0.155			
Previous PCI	0.747	0.235–2.379	0.622			
Previous stroke	1.701	0.376–7.704	0.491			
Creatinine, µmol/L	0.973	0.946–1.000	0.053	0.981	0.946–1.017	0.303
Hematocrit, g/L	0.998	0.967–1.030	0.901			
Hematocrit	0.992	0.889–1.108	0.891			
LAD lesion	0.032	0.004–0.239	0.001	0.032	0.004–0.238	0.001
Angiographic stenosis	1.010	0.970–1.051	0.636			

BMI, body mass index; PCI, percutaneous coronary intervention; LAD, left anterior descending artery; OR, odds ratio; CI, confidence interval.

**Table 6.** **Independent predictors of discordance between resting full-cycle ratio >0.89 and fractional flow reserve ≤0.80**.

	Univariate analysis			Multivariate analysis		
	OR	95% CI	*p-*value	OR	95% CI	*p-*value
Age, years	1.047	0.994–1.102	0.084	1.054	0.999–1.113	0.055
Female sex	1.141	0.484–2.691	0.762			
BMI, ${\mathrm{kg/m}}^{2}$	0.973	0.848–1.116	0.694			
Hypertension	0.783	0.335–1.826	0.571			
Dyslipidemia	1.920	0.721–5.113	0.192			
Diabetes	1.057	0.386–2.896	0.914			
Smoking	1.587	0.475–5.299	0.453			
Drinking	2.044	0.510–8.192	0.313			
Previous PCI	0.319	0.038–2.709	0.295			
Previous stroke	0.952	0.175–5.190	0.954			
Creatinine, µmol/L	0.977	0.947–1.008	0.144			
Hematocrit, g/L	0.974	0.941–1.008	0.130			
Hematocrit	0.944	0.836–1.065	0.349			
LAD lesion	0.462	0.183–1.170	0.103	0.406	0.155–1.059	0.065
Angiographic stenosis	1.033	0.971–1.098	0.304			

BMI, body mass index; PCI, percutaneous coronary intervention; LAD, left anterior descending artery; OR, odds ratio; CI, confidence interval.

Further analysis through multivariate logistic regression reinforced the importance of LAD location as an independent predictor of a discordance where RFR was ≤0.89 and FFR was >0.80 (odds ratio: 0.032, 95% confidence interval: 0.004–0.238; *p* < 0.01) (Table 5). However, when examining discordance within the gray zone (FFR: 0.75–0.80), the analysis did not reveal any independent predictors of discordance (**Supplementary Table 1**).

## 4. Discussion

Ischemic heart disease remains a major cause of cardiovascular morbidity and mortality worldwide [12]. Evidence-based medicine currently supports the use of FFR**-**guided revascularization strategies [13, 14], consistently reflected in revascularization guidelines [15, 16]. Despite this strong evidence and clear guidelines, the adoption of FFR–guided revascularization has been limited. This may be attributed to the requirement for vasodilators in measuring FFR, which can introduce complications and patient discomfort.

The emergence of NHPRs has enabled functional assessment without the need for vasodilators [10], thereby eliminating the related adverse effects and expanding functional assessment to a broader patient population. The iFR is a representative NHPR that operates at a precise timing during the diastole phase (“wave-free” period) [17]. Studies have shown that an iFR-guided revascularization is not inferior to FFR–guided approaches in terms of 1-year clinical outcomes [7, 18]. Another novel NHPR, RFR, has demonstrated a strong correlation and consistency with iFR in diagnosing functional stenosis in coronary arteries [5]. The validate RFR study highlighted that resting functional algorithms focusing only on the diastolic phase could potentially overlook vital data from the systolic phase [6]. Hence, RFR estimating coronary artery function during the entire cardiac cycle provides more accurate data and may offer greater clinical utility than the other NHPRs.

Our previous study reported a high degree of agreement between FFR and RFR, indicating the potential advantages of RFR [19]. This finding aligns with prior studies that also reported a good level of agreement between FFR and RFR in patients with coronary artery disease, where the diagnostic agreement rate ranged from 78.0% to 80.5% [8, 9, 10, 20, 21]. The current study further corroborates these findings by demonstrating a diagnostic agreement rate of 80.0%, thereby reinforcing the reliability and consistency of RFR alongside FFR in the assessment of coronary artery disease.

While our study demonstrated a high diagnostic agreement rate of 80.0% between FFR and RFR, an inconsistency of 20.0% remained in the evaluation of the diseased vessels. We categorized the 320 diseased vessels into four groups according to the FFR and RFR values. Our analysis revealed significant differences with lesions located in the LAD and degrees of vascular angiographic stenosis (*p* ≤ 0.001). The observed inconsistencies between FFR and RFR measurements under varying physiological conditions point to the need for further research. This future work should aim to elucidate the factors driving the discrepancies between these two diagnostic methods, enhancing our understanding and application of functional assessments in coronary artery disease.

Echoing our findings, a larger study involving 617 patients identified factors such as non-LCX lesions, the percentage of vessel stenosis diameter, and previous PCI of target vessels as independent predictors contributing to high RFR and low FFR [10]. In the study by Lee *et al*. [22], the percentage of vessel stenosis diameter was also a predictor of the inconsistency between high iFR and low FFR. All these studies provide valuable insights into the differences in degrees of vascular angiographic stenosis among the four groups. Our study noted that 56.9% of patients exhibited both negative FFR and RFR values. In clinical settings, functional coronary evaluation is often conducted to clarify the need for revascularization, especially when the degree of stenosis does not align with the patient’s clinical symptoms. This approach may explain the high negative prevalence of FFR and RFR in our population. Interestingly, the proportion of lesions with negative FFR and RFR in our study was closely aligned with results from a German Real-World Cohort study (56.9% vs. 55.6%) [10]. These observations underscore the importance of functional evaluation in managing coronary artery disease and the need for further research to understand the factors contributing to diagnostic inconsistencies.

In our investigation, the majority of diseased vessels were located in the LAD, accounting for 63.4% of the total. The study revealed varying degrees of consistency between FFR and RFR values, 74.0% (151/203) in LAD versus 90.0% (105/117) in the non-LAD areas. This distinction underscores a significantly lower consistency for LAD lesions compared to non-LAD lesions (*p* = 0.001), alongside a notable difference in FFR and RFR values between these two lesion locations (*p* < 0.001).

Previous studies have indicated that differences in the myocardial perfusion areas supplied by the left main artery, LAD, and non-LAD can affect the consistency between FFR and RFR [20]. Kato *et al*. [23] analyzed 410 patients (537 diseased vessels), and identified the LAD location, hemodialysis status, and female sex as independent predictors for discrepancies between low RFR and high FFR values, highlighting the potential overestimation of stenosis severity by RFR in patients undergoing hemodialysis or those with LAD disease. Similarly, Goto *et al*. [9] reported that LAD location, along with hemodialysis, diabetes, and anemia, were significant factors affecting the concordance between FFR and RFR in their study of 156 patients with 220 diseased vessels. They further emphasized LAD involvement as an independent predictor contributing to the diagnostic inconsistency between low RFR and high FFR (*p* < 0.01).

Further analysis of inconsistencies in the grey zone (FFR: 0.75–0.80) yielded no notable findings (**Supplementary Table 1**). Kobayashi *et al*. [24] speculated that the left main coronary artery in conjunction with the LAD provided a larger myocardial perfusion area compared to the non-LAD area, which may lead to more pronounced changes in the coronary artery flow between the resting and congested states, possibly explaining the inconsistency between FFR and iFR. Patients with LAD lesions exhibited higher resting coronary artery flow velocity and lower coronary flow reserve than those with non-LAD lesions [25]. An elevated resting coronary flow velocity results in a more substantial pressure step difference, which consequently results in lower RFR values. This could be one of the mechanisms contributing to the inconsistency observed between FFR and RFR, particularly in patients with LAD lesions.

Anatomical variations and location of the LAD can affect the measurement of FFR and RFR, leading to inconsistencies between the two metrics [20, 23]. First, the hemodynamic characteristics of LAD are affected by factors such as the anatomic length, curvature, branching point, and presence or absence of collateral circulation, these properties determine how blood vessels respond to pressure and their ability to regulate blood flow [26]. As a result, the anatomical diversity of LAD vessels can lead to diverging FFR and RFR values. Moreover, the specific location of lesions within the LAD can alter the measurement obtained from FFR and RFR. In FFR measurements, drug-induced vasodilation may lead to uneven pressure distribution in the distal vascular segment, affecting the FFR values [27]. On the other hand, RFR is mainly concerned with the change in the tube diameter during vasodilation and is not affected by drugs [6]. Weerts *et al*. [28] determined that distal lesion was an independent predictor of deferred target lesion failure. However, due to the lack of a detailed classification of LAD vessels at the beginning of the study design, unfortunately, we could not complete the study on the effect of the proximal, middle, and distal LAD vessels on the FFR and RFR consistency.

Future clinical studies are essential to validate these viewpoints. The impact of these specific characteristics should be considered in clinical practice when FFR diagnosis is inconsistent with RFR findings. When facing inconsistent FFR and RFR, it is important not to rely too heavily on a single physiological indicator. To improve the accuracy of diagnosis and treatment decisions, doctors should consider the patient’s overall condition, vascular anatomical characteristics, and lesion characteristics in their evaluations. Before measuring FFR, individualized drug preconditioning may be beneficial, especially for vascular segments known to exhibit variable responses to pharmacological agents. By adjusting for the physiological impacts of such drugs, the accuracy of FFR assessments can be improved [29]. Additionally, correcting for hydrostatic pressure offset has been shown to address specific consistency discrepancies between uncorrected and corrected values of major coronary artery branches, both at rest and under hyperemic conditions indicative of FFR [30]. Corrections based on hydrostatic pressure levels may thus be warranted. For patients whose conditions preclude definitive decision-making, ongoing clinical monitoring and periodic evaluations are advised. This approach enables the tracking of disease progression and the optimization of therapeutic outcomes, ensuring that treatment strategies are responsive to changes in the patient’s condition and the evolving nature of the disease.

## 5. Limitations

This study has several limitations that warrant consideration. First, it was a single-center, prospective registry study with a relatively modest cohort size, comprising 253 patients and 320 lesion vessels. This scale may limit the generalizability of the findings. Second, a significant proportion of the diseased vessels analyzed were located in the LAD, accounting for 63.4% of the diseased vessels. Given LAD’s extensive myocardial perfusion territory compared to non-LAD regions, this distribution could contribute to the observed discrepancies between RFR and FFR measurements. Additionally, our study included only 15 (5.9%) patients with NSTEMI. In real world settings, medical professionals found it easier to pinpoint the culprit lesions and target vessels in NSTEMI patients using CAG in conjunction with clinical indicators, potentially diminishing the need for functional assessment compared to UA patients. Furthermore, a preponderance of patients in our study underwent standard troponin testing instead of high-sensitivity troponin (hs-cTn) testing. Previous studies demonstrated that substituting hs-cTn testing for standard troponin testing enhances myocardial infarction detection rates (approximately 4% absolute and 20% relative increases), whereas UA diagnosis rates may drop in emergency department patients suspected of NSTE-ACS [11, 31, 32]. Lastly, the choice of adenosine triphosphate as a vasodilator, as opposed to the more commonly used adenosine in similar studies, may have implications in myocardial perfusion and coronary flow capacity, potentially affecting the correlation between RFR and FFR. These factors underscore the need for caution in extrapolating the study’s results and highlight areas for potential refinement in future studies.

## 6. Conclusions

The involvement of LAD has been identified as an independent predictor contributing to the diagnostic inconsistency between FFR and RFR in the functional assessment of coronary artery stenosis in patients with NSTE-ACS. This finding underscores the complex interplay between anatomical factors and physiological measurements in diagnosing coronary artery disease.

## Data Availability

The datasets used and/or analyzed during the current study are available from the corresponding author on reasonable request.

## Supplementary Material

Supplementary material associated with this article can be found, in the online version, at https://doi.org/10.31083/j.rcm2507260.
