# Supplementary material for: Comparison of Fractional Flow Reserve and Resting Full-Cycle Ratio in the Functional Assessment of Coronary Artery Stenosis in Patients with Non-ST-Segment Elevation Acute Coronary Syndrome
Source: Rev Cardiovasc Med. 2024 Jul 11;25(7):260. doi: 10.31083/j.rcm2507260 (PMC11317337; doi:10.31083/j.rcm2507260)
Supplement: Supplementary file 1 [file 2153-8174-25-7-260-s1.docx]

Supplementary Table 1. Independent predictors of discordance between the grey-zone (FFR: 0.75-0.80).

|  | Univariate analysis | | | Multivariate analysis | | |
| --- | --- | --- | --- | --- | --- | --- |
|  | **OR** | **95%CI** | ***p-*value** | **OR** | **95%CI** | ***p-*value** |
| Age, years | 0.969 | 0.906-1.038 | 0.369 |  |  |  |
| Female sex | 2.182 | 0.643-7.403 | 0.211 |  |  |  |
| BMI, kg/m^2^ | 1.058 | 0.871-1.286 | 0.571 |  |  |  |
| Hypertension | 0.583 | 0.169-2.014 | 0.394 |  |  |  |
| Dyslipidemia | 6.875 | 0.731-64.678 | 0.092 | 4.450 | 0.424-46.710 | 0.213 |
| Diabetes | 1.484 | 0.340-6.478 | 0.599 |  |  |  |
| Smoking | 5.176 | 0.529-50.653 | 0.158 |  |  |  |
| Previous stroke | 1.100 | 0.064-18.774 | 0.948 |  |  |  |
| Creatinine, μmol/L | 1.073 | 1.009-1.141 | 0.024 | 1.061 | 0.996-1.131 | 0.068 |
| Hematocrit, g/L | 1.034 | 0.991-1.078 | 0.119 |  |  |  |
| Hematocrit | 1.084 | 0.926-1.269 | 0.314 |  |  |  |
| LAD lesion | 1.400 | 0.366-5.350 | 0.623 |  |  |  |
| Angiographic stenosis | 0.929 | 0.852-1.012 | 0.093 | 0.959 | 0.871-1.055 | 0.390 |

BMI, body mass index; LAD, left anterior descending artery; FFR, fractional flow reserve; OR, odds ratio; CI, confidence interval.
